# A practical route to tertiary diarylmethylamides or -carbamates from imines, organozinc reagents and acyl chlorides or chloroformates

**DOI:** 10.3762/bjoc.7.112

**Published:** 2011-07-20

**Authors:** Erwan Le Gall, Antoine Pignon, Thierry Martens

**Affiliations:** 1 Électrochimie et Synthèse Organique, Institut de Chimie et des Matériaux Paris-Est, UMR 7182 CNRS - Université Paris-Est Créteil, 2-8 rue Henri Dunant, 94320 Thiais, France

**Keywords:** acyliminium, amides, carbamates, multicomponent reactions, organozinc reagents

## Abstract

A practical route to tertiary diarylmethylamides or -carbamates from imines, organozinc reagents and acyl chlorides or chloroformates is described. This route involves the formation of an imine, which is used without isolation, followed by its activation by the carbonyl-containing electrophile and the trapping of the acyliminium by an organozinc reagent. Most steps are conducted concomitantly to render the procedure as practical and straightforward as possible. Therefore, the whole experimental protocol takes less than two hours.

## Introduction

Diarylmethylamines constitute an important class of nitrogen-containing compounds displaying antihistaminic, anti-arrhythmic, diuretic, antidepressant, laxative, anesthetic and anticholinergic properties [1–2]. In this context, diarylmethylamides and -carbamates represent reliable *N*-protected diarylmethylamine derivatives and should thus serve as valuable precursors in the preparation of compounds of pharmaceutical interest. Several procedures enabling the construction of the diarylmethylamide and -carbamate core have been described. However, with respect to the substitution pattern of the expected final compound, available methods differ notably. Indeed, while the synthesis of secondary *N*-protected diarylmethylamines generally relies on the addition of organometallic reagents to electron-deficient (activated) imines [3–7], the preparation of tertiary diarylmethylamides or -carbamates may be conducted through the addition of aromatic nucleophiles onto *N*-acyliminium intermediates, formed in situ by reaction of imines with carbonyl-containing electrophiles. In this latter area, several studies have shown that some electron-enriched arenes can be used as nucleophiles and add efficiently onto the iminium carbon, either inter- or intramolecularly [8–16]. However, an increased range of aromatic moieties can be introduced through the use of organometallic compounds. The most commonly employed reagents are organoindium [17–18], organolithium [19–20], organomagnesium [21–22], organotin [23], or organozinc compounds [24–25]. However, although these are recognized as mild multi-purpose reagents, sole examples of their use in nucleophilic additions on acylimium salts consist, to the best of our knowledge, of the phenylation of quinolinium salts using diphenylzinc [26–27].

Recently, our group has been involved in various projects pertaining to the development of multicomponent reactions (MCRs) involving organometallic reagents, in particular organozinc reagents, due to their ability to react in very mild conditions and generally preserve most common functional groups. Moreover, used in stoichiometric amounts, organozinc reagents are more cost-effective and produce less toxic wastes than other common nucleophiles, such as, e.g., organoindium or organotin reagents. Our main contribution to the field was with regards to the use of arylzinc reagents in Mannich-type reactions with secondary amines and aldehydes to furnish tertiary diarylmethylamines [28–33]. However, while a large range of starting compounds could be used successfully in the process, we noticed that primary amines are ineffective, probably due to a weaker electrophilicity of the in situ-formed imines compared to iminium ions. Consequently, we report herein the use of primary amines in a sequential one-pot process, based on the preliminary activation of an aldimine with an acyl chloride or a chloroformate, and the subsequent trapping of the resulting acyliminium ion with an aromatic organozinc reagent, to generate a range of diarylmethylamides and -carbamates in satisfactory to good yields.

## Results and Discussion

**Scheme 1:**
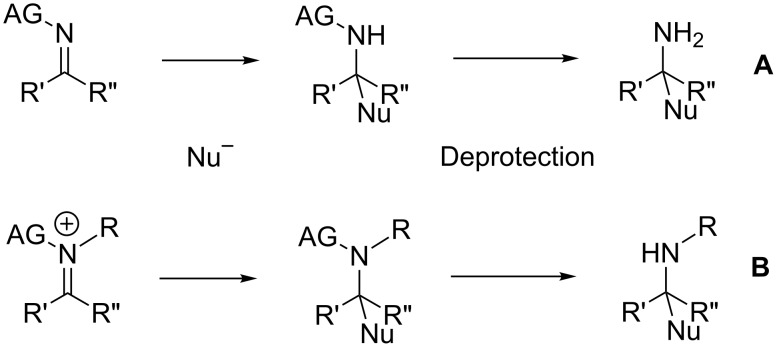
Addition of nucleophiles onto activated imines (**A**) or iminiums (**B**).

The limited intrinsic reactivity of imines towards the addition of nucleophiles has long been recognized as a major issue in nitrogen chemistry, but one which can be circumvented through several strategies, mainly intended to withdraw electrons and render the carbon more electrophilic [3–7]. Depending on the substitution pattern of the expected final amines, the increase of the electrophilicity should be implemented through the use of activated imines (Scheme 1, pathway **A**) or by quaternarization of the nitrogen atom with an electrophilic species (Scheme 1, pathway **B**). The activating group (AG) should then be released by a final deprotection to deliver the free amine (Scheme 1).

During the course of preceding works, we noticed that the addition of aromatic organozinc reagents onto *N*-substituted aldimines, formed in situ upon reaction of primary amines with aromatic aldehydes, cannot be undertaken under our established conditions. Thus, we intended to activate the C=N double bond by rendering the carbon more electrophilic and we initially envisaged the use of Lewis acid catalysis. Indeed, we assumed that under these conditions, the formation of N–AG (AG = activating group) bonds would be reversible, thus cleavage would be effective in situ and only relatively small amounts of the Lewis acid would be necessary. While several common Lewis acids (TiCl_4_, AlCl_3_, CeCl_3_ and BF_3_·Et_2_O) were trialled unsuccessfully, a different strategy based on the formation of solid bonds indicated that carbonyl derivatives such as acetyl chloride or methyl chloroformate were, in contrast, efficient activators of the C=N double bond, albeit used in stoichiometric amounts. This result is consistent with some previous studies reporting the activation of imines under an acyliminium form and the subsequent addition of either aromatic [17–23,26–27] or non-aromatic [34–35] organometallic nucleophiles onto carbon.

Our preliminary investigations were then conducted on *N*-benzylidenepropan-1-amine, taken as a model aldimine, which was preformed and purified prior to use. This compound was subjected to consecutive reactions with acetyl chloride and phenylzinc bromide, furnishing the corresponding diarylmethylamide in good yield (80%). However, as supplementary experiments indicated that the starting imine **1** can be used without preliminary purification, we chose to simplify the process by operating from the crude imine, although slightly lower yields (10–15% decreasing) were hence obtained. Thus, in a typical experiment, the amine and the aldehyde were heated in toluene in the presence of 4 Å molecular sieves for a few minutes. After cooling to room temperature, the toluene solution containing the imine **1** was transferred into another flask in which a slight excess of the electrophile (acyl chloride or chloroformate **2**) was added. After a limited period under heating, the arylzinc reagent **3**, prepared in parallel via a cobalt-catalyzed procedure [36] was added and the resulting solution was stirred for 30 minutes at ambient temperature. The chromatographic purification of the crude oil afforded the expected diarylmethylamide or -carbamate **4**. Representative experimental results are reported in Table 1.

**Table 1:** Formation of diarylmethylamides and -carbamates.^a^

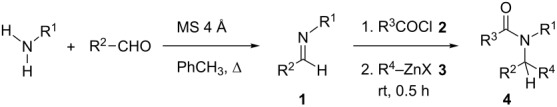						

Entry	R^1^	R^2^	R^3^	R^4^	Product	Isolated yield (%)

1	*n*-Pr	Ph	Me	Ph	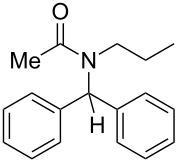 **4a**	64
2	*n*-Pr	3-O_2_N–C_6_H_4_–	Me	Ph	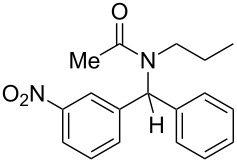 **4b**	55
3	*n*-Pr	3-O_2_N–C_6_H_4_–	MeO	Ph	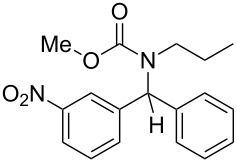 **4c**	59
4	*n*-Pr	2-F_3_C–C_6_H_4_–	MeO	Ph	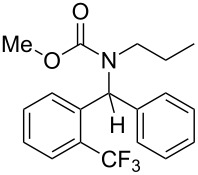 **4d**	67
5	*n*-Pr	thiophen-3-yl	MeO	Ph	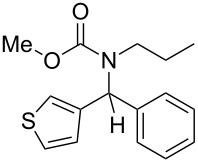 **4e**	42
6	*n*-Pr	Ph	MeO	3-F_3_C–C_6_H_4_–	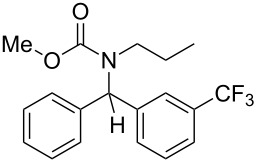 **4f**	68
7	*n*-Pr	Ph	MeO	4-EtO_2_C–C_6_H_4_–	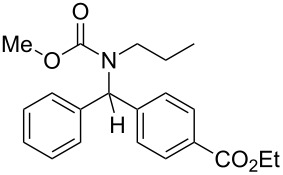 **4g**	57
8	*n*-Pr	Ph	MeO	4-Cl–C_6_H_4_–	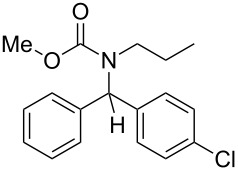 **4h**	71
9	*n*-Pr	Ph	MeO	4-MeO–C_6_H_4_–	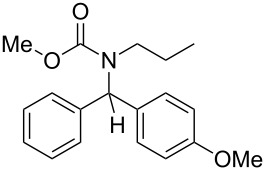 **4i**	63
10	Ph	Ph	Me	Ph	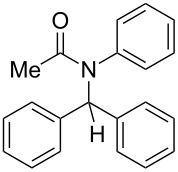 **4j**	69
11	Bn	Ph	Me	Ph	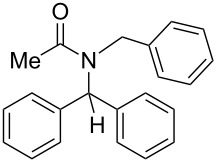 **4k**	36

^a^Experiments were conducted with ~10 mmol of imine, 12 mmol of acyl chloride or chloroformate, 13–16 mmol of the organozinc reagent, prepared from 20 mmol of aryl bromide.

Under these conditions, coupling products **4** are formed in low to high yields. The use of acetyl chloride (Table 1, entries 1, 2, 10 and 11) provided similar results to those observed with methyl chloroformate (Table 1, entries 3–9). It can be seen that more limited yields were obtained when a thiophene-derived aldehyde (Table 1, entry 5) or benzylamine was employed as the starting amine (Table 1, entry 11). However, these last two results could not be explained.

**Scheme 2:**
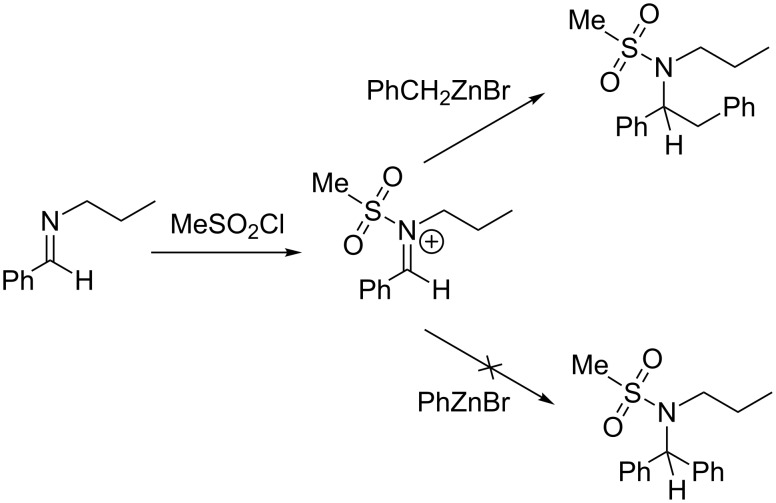
Activation of the aldimine with MsCl.

We next tried to extend the reaction to other electrophilic compounds that are known to easily form N–AG bonds with imines and furnish analogous iminium salts. The case of methanesulfonyl chloride (MsCl) was dealt with first (Scheme 2).

Unfortunately, while the reaction of benzylzinc bromide proved efficient under MsCl activation, phenylzinc bromide did not undergo the coupling at all [37]. This was also the case with trimethylsilyl chloride as an activator, whose reaction with the model aldimine and phenylzinc bromide did not afford the expected compound. On the other hand, a preliminary experiment indicated that trifluoracetic anhydride was a very reliable activator of the imine towards phenylzinc bromide addition. These results, combined with the above reported observations with common Lewis acids, may indicate that acylating reagents are particularly reliable for the activation of aldimines toward arylzinc additions. However, the use of carbonyl-containing electrophiles obviously constitutes an important drawback of the procedure. Indeed, although TBAF has been reported to constitute a mild deprotection reagent for a range of carbamates [38–39], the cleavage of the N–C=O bond, which is formed during the process, might be commonly achieved under rather harsh conditions. This is not the case with other potential activating agents such as sulfinic or phosphinic chloride derivatives (ClS(O)R and ClP(O)R_2_), whose N–AG bond might be cleaved easily upon acidic work-up. In addition, chiral versions of such activators would be of further interest for potential asymmetric couplings (at least with benzylzinc reagents), as the stereogenic center would be located very close to the impending asymmetric carbon and may thus serve as a valuable chiral auxiliary. Consequently, we envisage the implementation a further study which would be dedicated to the evaluation of well-recognized chiral inductors such as Ellman- [40–41] or Davis-type [42–43] sulfinyl derivatives in the process.

## Conclusion

In conclusion, the results reported in this study indicate that the formation of acyliminium cations constitutes a very convenient approach to the activation of imines toward the addition of aromatic organozinc reagents. Indeed, we could prepare a range of diarylmethylamides or diarylmethylcarbamates by a sequential multicomponent process involving the preliminary formation of an imine, which can be used without isolation, its activation by an acyl chloride or a chloroformate and the final trapping of the resulting acyliminium salt by an arylzinc reagent. However, the harsh conditions which would probably be required for the deprotection of the amide or carbamate function prompt us to undertake complementary experiments dedicated to the assessment of easier-to-cleave activating groups. Consequently, the evaluation of sulfinyl- or phosphinyl derivatives in the process has been undertaken recently and will be reported in due course.

## Experimental

### Typical procedure for the preparation of diarylmethylamides and carbamates

The aldimine (~10 mmol) was prepared from the aromatic aldehyde (12 mmol) and the amine (12 mmol) in toluene (10 mL) in the presence of 4 Å molecular sieves (10 g) and *para*-toluenesulfonic acid (10 mg). After 30 min stirring at 80 °C and cooling to rt, the solution was taken-up with a syringe and the sieves washed with 5 mL toluene. The combined toluene fractions were placed in another flask, which was flushed with argon prior to addition, and acetyl chloride or methyl chloroformate (12 mmol) was added. The resulting mixture was stirred at rt (ClCOCH_3_) or at 50 °C (ClCOOCH_3_) for 30 min, a period during which the aromatic organozinc reagent (13–16 mmol, depending on the starting halide) was prepared concomitantly as follows: A 100 mL round bottom flask was flushed with argon, then acetonitrile (20 mL), zinc dust (3 g), TFA (0.2 mL) and BrCH_2_CH_2_Br (0.2 mL) were added consecutively under vigorous (~500 rpm) stirring. The mixture was heated until gas was evolved (at 50–70 °C), then allowed to cool to rt under continuous stirring. The aryl bromide (15 mmol) and anhydrous cobalt bromide (330 mg) were then added to the mixture, which was stirred at rt for additional 20 min. Stirring was then stopped and the surrounding solution was taken-up with a syringe. The solution was then added to the flask containing the imine/carbonyl-containing compound mixture and the resulting mixture was stirred at rt for 30 min. The solution was poured into a sat. NH_4_Cl solution (100 mL), extracted with diethyl ether (2 × 75 mL) and the combined organic fractions were dried with magnesium sulfate, filtrated and then concentrated under reduced pressure. The crude oil was purified by column chromatography over silica gel with a pentane/diethyl ether mixture (1:0 to 0:1) as an eluant to afford the diarylmethylamide or carbamate **4**.

#### NMR data for selected compounds

Methyl phenyl(2-(trifluoromethyl)phenyl)methyl(propyl)carbamate (**4d**) ^1^H NMR (400 MHz, CDCl_3_) δ 7.73 (d, *J* = 7.8 Hz, 1H), 7.51 (t, *J* = 7.5 Hz, 1H), 7.43 (t, *J* = 7.6 Hz, 1H), 7.37–7.25 (m, 4H), 7.12 (d, *J* = 7.2 Hz, 2H), 6.94 (s, 1H), 3.71 (s, 3H), 3.45–3.31 (m, 1H), 3.23–3.11 (m, 1H), 1.27–1.11 (m, 1H), 0.79 (br s, 1H), 0.56 (t, *J* = 7.4 Hz, 3H); ^13^C NMR (100 MHz, CDCl_3_) δ 156.9, 139.9, 139.5, 131.7, 131.0, 129.5 (q, *J* = 30.3 Hz), 128.4, 128.0, 127.9, 127.4, 126.4 (q, *J* = 6.0 Hz), 124.2 (q, *J* = 274.4 Hz), 59.4, 52.7, 47.5, 21.9, 11.11.

*N*-Benzhydryl-*N*-phenylacetamide (**4j**) ^1^H NMR (400 MHz, CDCl_3_) δ 7.19–7.10 (m, 15H), 6.74 (s, 1H), 1.86 (s, 3H); ^13^C NMR (100 MHz, CDCl_3_) δ 170.8, 140.9, 139.2, 130.2, 129.7, 128.9, 128.1, 128.0, 127.4, 64.1, 23.7.

*N*-Benzhydryl-*N*-benzylacetamide (**4k**) ^1^H NMR (400 MHz, CDCl_3_) δ 7.15–6.96 (m, 14H), 6.67–6.64 (m, 2H), 4.57 (s, 2H), 2.04 (s, 3H); ^13^C NMR (100 MHz, CDCl_3_) δ 172.2, 139.3, 137.4, 129.2, 128.5, 128.2, 127.9, 127.6, 125.8, 66.4, 48.0, 22.8.

## References

[1] Spencer C M, Faulds D, Peters D H (1993). Drugs.

[2] Sakurai S, Ogawa N, Suzuki T, Kato K, Ohashi T, Yasuda S, Kato H, Ito Y (1996). Chem Pharm Bull.

[3] Enders D, Reinhold U (1997). Tetrahedron: Asymmetry.

[4] Bloch R (1998). Chem Rev.

[5] Kobayashi S, Ishitani H (1999). Chem Rev.

[6] Friestad G K, Mathies A K (2007). Tetrahedron.

[7] Hermanns N, Dahmen S, Bolm C, Bräse S (2002). Angew Chem.

[8] Zhang Y, Kindelin P J, DeSchepper D J, Zheng C, Klumpp D A (2006). Synthesis.

[9] Kitabatake M, Saitoh T, Sano T, Horiguchi Y (2009). Heterocycles.

[10] Zhang Y, DeSchepper D J, Gilbert T M, Sai K K S, Klumpp D A (2007). Chem Commun.

[11] Romero M, Caignard D-H, Renard P, Pujol M D (2008). Tetrahedron.

[12] Klumpp D A, Zhang Y, O’Connor M J, Esteves P M, de Almeida L S (2007). Org Lett.

[13] Venkov A P, Statkova-Abeghe S (1996). Synth Commun.

[14] Venkov A P, Statkova S M, Ivanov I I (1992). Synth Commun.

[15] Venkov A P, Statkova S M (1991). Synth Commun.

[16] Venkov A P, Mollov N M (1982). Synthesis.

[17] Black D A, Arndtsen B A (2006). Org Lett.

[18] Beveridge R E, Black D A, Arndtsen B A (2010). Eur J Org Chem.

[19] Lamblin M, Couture A, Deniau E, Grandclaudon P (2008). Tetrahedron: Asymmetry.

[20] Alexakis A, Amiot F (2002). Tetrahedron: Asymmetry.

[21] Venkov A P, Statkova-Abeghe S M (1995). Synth Commun.

[22] Pegoraro S, Lang M, Dreker T, Kraus J, Hamm S, Meere C, Feurle J, Tasler S, Prütting S, Kuras Z (2009). Bioorg Med Chem Lett.

[23] Black D A, Arndtsen B A (2005). J Org Chem.

[24] Knochel P, Singer R D (1993). Chem Rev.

[25] Knochel P, Jones P (1999). Organozinc Reagents, A Practical Approach.

[26] Ludwig M, Polborn K, Wanner K T (2003). Heterocycles.

[27] Bender C, Liebscher J (2009). ARKIVOC.

[28] Haurena C, Le Gall E, Sengmany S, Martens T (2010). Tetrahedron.

[29] Le Gall E, Haurena C, Sengmany S, Martens T, Troupel M (2009). J Org Chem.

[30] Sengmany S, Le Gall E, Troupel M (2008). Synlett.

[31] Sengmany S, Le Gall E, Le Jean C, Troupel M, Nédélec J-Y (2007). Tetrahedron.

[32] Le Gall E, Troupel M, Nédélec J-Y (2006). Tetrahedron.

[33] Le Gall E, Troupel M, Nédélec J-Y (2006). Tetrahedron Lett.

[34] Black D A, Arndtsen B A (2004). Org Lett.

[35] Fischer C, Carreira E M (2004). Org Lett.

[36] Fillon H, Gosmini C, Périchon J (2003). J Am Chem Soc.

[R37] 37The more important reactivity of benzylzinc vs arylzinc reagents has been noticed on several occasions. For instance, we have already shown that benzyl bromides react, under Barbier-like conditions, with aldehydes and primary amines whereas aryl bromides do not undergo the coupling at all. This result is consistent with a possible nucleophilic addition of benzylzinc reagents onto imines without mandatory activation of the latter. See ref. [29] for details.

[38] Routier S, Saugé L, Ayerbe N, Coudert G, Mérour J-Y (2002). Tetrahedron Lett.

[39] Jacquemard U, Bénéteau V, Lefoix M, Routier S, Mérour J-Y, Coudert G (2004). Tetrahedron.

[40] Liu G, Cogan D A, Ellman J A (1997). J Am Chem Soc.

[41] Ellman J A, Owens T D, Tang T P (2002). Acc Chem Res.

[42] Davis F A (2006). J Org Chem.

[43] Zhou P, Chen B-C, Davis F A (2004). Tetrahedron.

